# Associations Among Demographic Characteristics, Sleep Behaviors, Physical Activity, Health Status, and Quality of Life Among Undergraduate Students: A Structural Equation Modeling Study

**DOI:** 10.3390/healthcare14162528

**Published:** 2026-08-13

**Authors:** Bandar S. Alharbi, Majed M. Aljabri

**Affiliations:** Community and Psychiatric Mental Health Nursing Department, College of Nursing, King Saud University, Riyadh 12375, Saudi Arabia; banalharbi@ksu.edu.sa

**Keywords:** quality of life, university students, structural equation modeling, sleep quality, physical activity, psychological distress, DASS-21, WHOQOL-BREF

## Abstract

**Background**: Quality of life (QoL) among university students is an important indicator of overall health and academic success. Although these factors are interrelated, few studies have simultaneously examined their structural associations with QoL among university students in Saudi Arabia. This study investigated the associations among demographic characteristics, lifestyle behaviors, psychological distress, and quality of life using structural equation modeling (SEM). **Methods**: We conducted a cross-sectional study among undergraduate students enrolled in public universities in Riyadh, Saudi Arabia. The survey included demographic characteristics, the Depression Anxiety Stress Scale-21 (DASS-21), the World Health Organization Quality of Life-BREF (WHOQOL-BREF), and measures of sleep quality, sleep duration, and physical activity. Confirmatory factor analysis was performed to evaluate the measurement model. We fitted SEM to examine associations among demographic characteristics, lifestyle behaviors, health status, and quality of life. **Results:** A total of 198 undergraduate students were included in the analysis. The measurement model demonstrated satisfactory psychometric properties. The SEM demonstrated acceptable model fit (χ^2^/df = 2.00, Comparative Fit Index = 0.937, Tucker–Lewis Index = 0.914, Root Mean Square Error of Approximation = 0.071, Standardized Root Mean Square Residual = 0.070). Male students reported poorer sleep quality (β = −0.405, *p* < 0.001), shorter sleep duration (β = −0.208, *p* = 0.019), and lower physical activity (β = −0.299, *p* < 0.001) than female students. Students with a GPA of 4.50 or above reported higher physical activity than those with lower GPAs (β = 0.182, *p* = 0.027). Better sleep quality (β = 0.169, *p* = 0.024) and very good or excellent self-rated health (β = 0.492, *p* < 0.001) were positively associated with quality of life, whereas sleep duration was not significantly associated with quality of life (*p* = 0.225). **Conclusions**: Quality of life among undergraduate students was associated with multiple interconnected demographic and lifestyle factors. Our findings highlight the importance of integrated university health-promotion strategies that simultaneously address sleep health, healthy lifestyle behaviors, and psychological well-being to improve students’ overall quality of life. Longitudinal studies should be conducted to clarify the temporal relationships among these factors.

## 1. Introduction

University life represents a critical developmental period characterized by substantial academic, psychological, and social transitions that can profoundly influence students’ health and well-being [1,2]. This is because, at this stage, students are expected to adapt to increased academic demands, greater personal responsibility, financial pressures, changing living arrangements, and new social environments while simultaneously establishing lifelong health behaviors. Although these experiences promote personal growth, they may also increase vulnerability to unhealthy lifestyles, psychological distress, and diminished quality of life (QoL) [3,4]. This means promoting students’ well-being is an important public health and educational priority.

The World Health Organization (WHO) defines quality of life as individuals’ perception of their position in life within the context of their culture, value systems, goals, expectations, and concerns [5]. Quality of life shows a multidimensional construct that includes physical health, psychological well-being, social relationships, and environmental conditions. Previous evidence indicates that university students frequently report lower QoL than the general young adult population due to academic stress, irregular lifestyles, inadequate sleep, reduced physical activity, and increasing mental health challenges [4,6]. On the other hand, these factors rarely occur independently but instead interact in complex ways, making comprehensive evaluation essential.

Psychological distress has become one of the most prevalent health concerns among university students worldwide [7]. Depression, anxiety, and stress are increasingly recognized as common experiences during higher education, affecting emotional well-being, concentration, interpersonal relationships, and academic performance [8]. This can be related to the fact that transition to university often coincides with greater academic competition, financial concerns, and reduced family support, all of which may contribute to elevated psychological distress. Several studies have consistently showed that higher levels of depression, anxiety, and stress are associated with poorer health-related quality of life across physical, psychological, and social domains [9,10,11]. On the other hand, psychological distress may negatively influence lifestyle behaviors, including sleep habits and physical activity, which may result in interconnected pathways through which mental health affects overall well-being.

Sleep represents another fundamental determinant of health during young adulthood. Adequate sleep is essential for cognitive functioning, emotional regulation, memory consolidation, immune function, and academic performance [12]. Nevertheless, university students frequently experience inadequate sleep duration and poor sleep quality because of academic workload, excessive screen exposure, irregular schedules, and social commitments. Students’ overall well-being can also be affected by regular physical activity. Physical activity contributes to cardiovascular fitness, metabolic health, stress reduction, emotional resilience, and cognitive functioning [13]. Evidence also suggests that physically active students report better self-rated health, fewer depressive symptoms, higher life satisfaction, and superior quality of life than their less-active peers [14].

In Saudi Arabia, growing attention has been directed toward student mental health and well-being in response to increasing recognition of psychological distress and unhealthy lifestyle behaviors among university students [15]. Nevertheless, much of the existing literature has focused on individual outcomes, such as depression, anxiety, sleep quality, or physical activity, using conventional regression approaches. Few studies have simultaneously examined how these factors interact to influence overall quality of life, particularly among students attending public universities.

Structural equation modeling (SEM) provides an appropriate analysis framework to investigate these complex associations [16]. It enables the simultaneous estimation of multiple direct associations while accounting for measurement error through latent constructs. Unlike traditional regression models, SEM allows psychological distress and quality of life to be represented as multidimensional latent variables, thereby providing a more comprehensive understanding of the relationships among demographic characteristics, lifestyle behaviors, mental health, and overall well-being.

Therefore, we aimed to examine the structural associations among demographic characteristics, sleep quality, sleep duration, physical activity, self-rated health, psychological distress, and quality of life among undergraduate students enrolled in public universities in Riyadh, Saudi Arabia. Psychological distress was modeled as a latent construct based on depression, anxiety, and stress symptoms. By simultaneously modeling these interrelated factors using SEM, this study aims to provide a more comprehensive understanding of the determinants of students’ quality of life.

## 2. Methods

### 2.1. Study Design

We conducted a cross-sectional study design using a questionnaire-based study among undergraduate students enrolled in three public universities in Riyadh, Saudi Arabia. A convenience sampling strategy was employed to recruit students from participating public universities. Data were collected over a two-week period between 10 June and 25 June 2026 using a self-administered online questionnaire developed in Google Forms. Recruitment was conducted through campus-wide announcements displayed on university notice boards. The announcements contained a brief description of this study, the eligibility criteria, information regarding voluntary participation, and a Quick Response (QR) code that directed interested students to the online questionnaire. Students accessed the survey by scanning the QR code using their mobile devices. Because the survey was anonymous and completed electronically, no personally identifiable information was collected. The distribution of participants across universities reflected voluntary participation rather than proportional institutional enrollment.

### 2.2. Participants and Sampling

Eligible participants were undergraduate students aged 18 years or older who were enrolled in one of the participating public universities in Riyadh during the study period. Students who were unable to understand Arabic or English or who submitted incomplete questionnaires with substantial missing information were excluded from the analysis. Participation was entirely voluntary. Before accessing the questionnaire, participants were presented with an electronic information sheet describing the study objectives, expected completion time, confidentiality procedures, and their right to withdraw from this study at any time before submission. Electronic informed consent was obtained by asking participants to indicate their agreement before proceeding to the questionnaire. A total of 229 students accessed the online questionnaire. Thirty-one questionnaires were excluded because they were incomplete and lacked responses to one or more of the principal study variables required for the structural equation modeling analysis. Consequently, data from 198 participants were included in the final analysis.

Data collection procedure: Data were collected using Google Forms, an online survey platform that enables secure electronic data capture. The survey link was embedded within a QR code displayed on university announcement boards across participating campuses. To reduce duplicate responses, participants were allowed to submit the questionnaire only once per device through Google Forms settings.

### 2.3. Measures

We collected data on students’ demographic and academic characteristics, including age group, sex, living arrangement, academic year, current grade-point average (GPA), learning modality, self-rated health status, average sleep duration, perceived sleep quality, primary source of financial support, physical activity frequency, and part-time employment status. These variables were measured using structured categorical response options developed specifically for this study. Age was categorized into three groups (18–20, 21–23, and ≥24 years). The original age categories were reviewed during data preparation, and the 24–26- and ≥27-year groups were combined because the oldest category contained a small number of participants. This recategorization was performed to ensure adequate sample sizes within each category and to improve the stability and interpretability of the statistical analyses. GPA, health status, sleep quality, sleep duration, and physical activity were assessed using ordered categorical response scales.

We used the original English version of Depression Anxiety Stress Scale–21 items (DASS-21) to assess psychological distress, which was originally developed by Lovibond and Lovibond (1995) as a shortened version of the 42-item DASS [17]. The instrument comprises 21 items grouped into three seven-item subscales measuring depression, anxiety, and stress. Each item assesses symptoms experienced during the previous week and is rated on a four-point Likert scale ranging from 0 (“Did not apply to me at all”) to 3 (“Applied to me very much or most of the time”). Subscale scores are calculated by summing the seven corresponding items and multiplying the total by two to obtain scores comparable to the original DASS-42. Higher scores indicate greater psychological distress. Severity classifications (normal, mild, moderate, severe, and extremely severe) were derived according to the recommended DASS-21 cut-off values. In our study, the depression, anxiety, and stress subscales served as observed indicators of the latent construct Mental Distress in the SEM.

Quality of life was measured using the English version World Health Organization Quality of Life-BREF (WHOQOL-BREF) developed by the WHOQOL Group (1998) [18]. The WHOQOL-BREF is a widely used generic health-related quality of life instrument consisting of 26 items that evaluate four domains including physical health, psychological health, social relationships, and environment. There are also two additional items used to assess overall quality of life and overall satisfaction with health. Items are scored on five-point Likert scales, with response categories varying according to item wording. Negatively phrased items are reverse-scored before domain scores are calculated. Domain scores are transformed to either a 4–20 scale or a standardized 0–100 scale, with higher scores indicating better quality of life. The WHOQOL-BREF has been translated into more than 40 languages and demonstrates good cross-cultural validity, including validated Arabic versions used among university students and general adult populations. In this study, we modeled the four WHOQOL-BREF domain scores as indicators of the latent construct Quality of Life.

Sleep quality was assessed using a single self-reported question asking participants to rate their overall sleep quality on a five-category ordinal scale ranging from very poor to very good. Average sleep duration was measured using a single question asking participants to report the average number of hours slept per day (<5 h, 5–7 h, or >7 h). These measures have been widely used in epidemiological studies because they provide practical indicators of habitual sleep behavior while minimizing participant burden.

Physical activity was assessed using a single-item measure asking participants to report the frequency of engaging in physical activity or exercise. Responses ranged from never to five or more times per week using four ordered response categories.

### 2.4. Statistical Analysis

We used mean and standard deviation to summarize the descriptive statistics for continuous variables, whereas categorical variables were presented as frequencies and percentages.

Confirmatory factor analysis (CFA) was used to evaluate the measurement properties of the latent constructs representing Mental Distress and Quality of Life. Internal consistency was assessed using Cronbach’s alpha and composite reliability (CR), while convergent validity was evaluated using the average variance extracted (AVE). Standardized factor loadings greater than 0.50, CR values ≥ 0.70, and AVE values ≥ 0.50 were considered indicative of acceptable measurement properties.

Structural equation modeling (SEM) was subsequently employed to examine the associations between demographic characteristics, lifestyle behaviors, health status, and quality of life. Demographic variables were entered as exogenous predictors, while sleep quality, sleep duration, and physical activity were modeled as endogenous lifestyle variables. Quality of life was specified as the final endogenous latent outcome. Psychological distress was specified as a latent measurement construct, with depression, anxiety, and stress symptoms as its observed indicators. The construct was included to represent the shared underlying dimension of psychological distress but was not specified as a structural predictor of quality of life in the final model.

Categorical predictors with more than two categories were dummy-coded using meaningful reference groups prior to analysis to improve interpretability of the regression coefficients. Parameters were estimated using the robust maximum likelihood (MLR) estimator with 5000 bootstrap resamples to obtain robust standard errors and bias-corrected 95% confidence intervals.

Model fit was evaluated using multiple complementary fit indices following widely accepted recommendations. Adequate model fit was defined as a Comparative Fit Index (CFI) and Tucker–Lewis Index (TLI) ≥ 0.90, Root Mean Square Error of Approximation (RMSEA) ≤ 0.08, and Standardized Root Mean Square Residual (SRMR) ≤ 0.08. Standardized regression coefficients (β), 95% confidence intervals, and corresponding *p*-values were reported for all structural associations.

All statistical analyses were performed in R version 4.4.3 [19].

## 3. Results

Table 1 summarizes the characteristics of 198 undergraduate university students. About 57.8% of the students were aged 21–23 years old and 22.2% were aged 18–20 years. A total of 53.0% of participants were male, and most lived with their families (59.6%), whereas 34.3% resided in university housing and 6.1% reported other living arrangements (living alone or with friends). About 43.9% of students were in their third year of study, followed by 23.2% in the second year and 23.2% in the fourth year or internship, while only 9.6% were first-year students. Approximately half of the participants (49.0%) reported a grade-point average (GPA) between 3.50 and 4.49, with 20.2% achieving a GPA of 4.50–5.00.

Most students perceived their health as good (50.5%) or very good/excellent (28.3%), whereas 21.2% rated their health as poor or fair. Sleep quality was predominantly rated as fair (41.9%), followed by good (31.3%) and poor (26.8%). More than half of the participants (54.5%) reported sleeping 5–7 h per night, while 30.3% slept more than seven hours and 15.2% slept less than five hours. Nearly half of the students (49.5%) engaged in physical activity 1–2 times per week, whereas 35.4% exercised three or more times weekly, and 15.2% reported no regular physical activity. Approximately one-quarter (26.8%) were engaged in part-time employment. The majority (85.4%) attended face-to-face learning, with relatively few participating in blended (8.1%) or online (6.6%) learning modalities.

The mean (SD) scores for the Depression, Anxiety, and Stress Scale (DASS-21) were 16.0 (8.0) for depression, 16.0 (8.0) for anxiety, and 17.0 (7.0) for stress. The mean WHOQOL-BREF domain scores were 11.81 (2.29) for physical health, 11.63 (2.57) for psychological health, 10.60 (3.80) for social relationships, and 11.30 (3.10) for the environmental domain.

**Figure 1:**
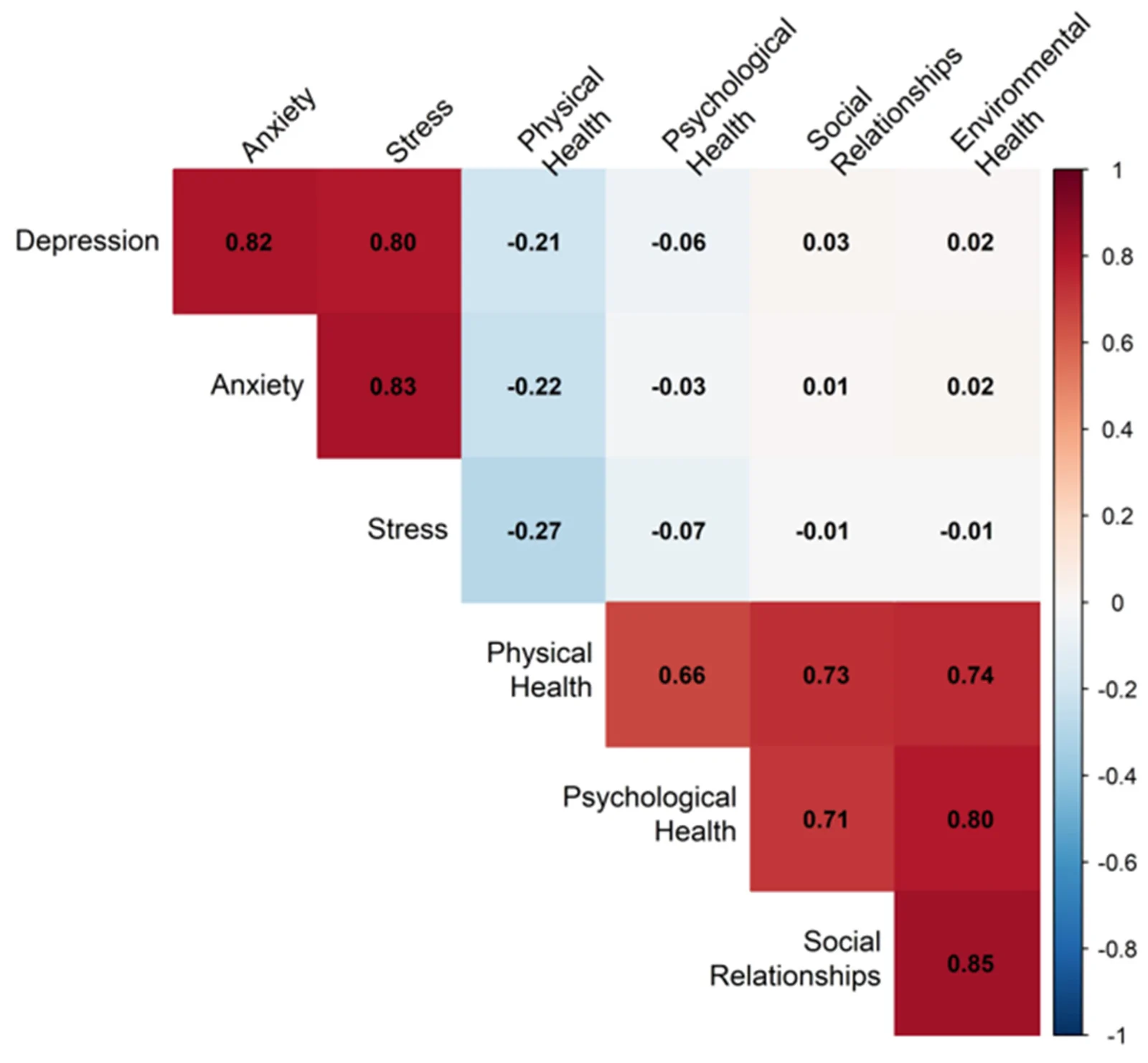
Pearson’s correlation matrix of psychological distress and quality of life domains among undergraduate students.

Figure 1 presents the Pearson correlation matrix for the psychological distress and quality of life domains. The three psychological distress dimensions, depression, anxiety, and stress, were positively correlated, with correlation coefficients ranging from 0.797 to 0.830. Similarly, the WHOQOL-BREF domains demonstrated moderate-to-strong positive intercorrelations (r = 0.664–0.848). This suggests that better quality of life in one domain was generally associated with better quality of life across the other domains. In contrast, depression, anxiety, and stress exhibited weak negative correlations with the physical health (r = −0.208 to −0.272) and psychological health (r = −0.030 to −0.068) domains of quality of life. This means that higher levels of psychological distress were associated with lower perceived physical and psychological well-being. Correlations between psychological distress and the social relationships (r = −0.005 to 0.028) and environmental (r = −0.005 to 0.023) domains were negligible. The correlation pattern supports the conceptual distinction between psychological distress and quality of life while demonstrating strong within-construct associations. It provides preliminary evidence for the measurement model evaluated in the subsequent structural equation modeling analyses.

The DASS-21 demonstrated acceptable-to-good internal consistency, with Cronbach’s alpha coefficients ranging from 0.75 to 0.81 and McDonald’s omega coefficients ranging from 0.83 to 0.86 across the depression, anxiety, and stress subscales (Supplementary Table S1). The WHOQOL-BREF also showed acceptable-to-excellent reliability, with Cronbach’s alpha values ranging from 0.72 to 0.87 and omega coefficients ranging from 0.81 to 0.90. The environmental domain demonstrated the highest internal consistency (α = 0.87, ω = 0.90), whereas the psychological health domain showed comparatively lower reliability (α = 0.72). The reliability test indicated satisfactory internal consistency of the study instruments for subsequent correlation, regression, and structural equation modeling analyses. All standardized factor loadings were statistically significant (*p* < 0.001) and ranged from 0.794 to 0.952 (Supplementary Table S2). The Mental Distress construct exhibited excellent internal consistency (Cronbach’s α = 0.928, CR = 0.929, AVE = 0.814), while the Quality of Life construct also demonstrated excellent reliability (Cronbach’s α = 0.911, CR = 0.929, AVE = 0.778). All AVE values exceeded the recommended threshold of 0.50. This means that each latent construct explained more than half of the variance in its observed indicators and supporting adequate convergent validity.

Our structure equation model showed an acceptable overall fit to the data (Table 2). The chi-square statistic was significant (χ^2^ = 168.13, df = 84, *p* < 0.001), which is common in SEM because the test is sensitive to sample size. Incremental fit indices also supported adequate model fit, with a Comparative Fit Index (CFI) of 0.937 and a Tucker–Lewis Index (TLI) of 0.914, both exceeding the recommended threshold of 0.90. Absolute fit indices further suggested acceptable fit, with a Root Mean Square Error of Approximation (RMSEA) of 0.071 (90% CI: 0.055–0.087) and a Standardized Root Mean Square Residual (SRMR) of 0.070. The indices overall indicate that the proposed structural equation model provided an acceptable representation of the observed data.

The structural model identified several significant associations between demographic characteristics, lifestyle behaviors, and quality of life (Table 3). We found that, compared with female students, male students showed significantly poorer sleep quality (β = −0.405, *p* < 0.001), shorter sleep duration (β = −0.208, *p* = 0.019), and lower levels of physical activity (β = −0.299, *p* < 0.001). We did not find a significant association between age group, living arrangement, GPA, or financial support and sleep quality. Similarly, age group, living arrangement, GPA, and financial support were not significantly associated with sleep duration, although students living in residences other than the family home showed a marginally lower sleep duration than those living with their families (β = −0.114, *p* = 0.056), and students reporting low financial support demonstrated a marginally longer sleep duration than those with high financial support (β = 0.120, *p* = 0.078). For physical activity, students with a GPA of 4.50 or higher showed significantly higher physical activity levels than those with a GPA below 3.50 (β = 0.182, *p* = 0.027). No significant associations were observed for age group, living arrangement, or financial support. For quality of life on the other hand, students reporting very good or excellent health status had significantly higher quality of life than those reporting poor or fair health (β = 0.492, *p* < 0.001). In addition, better sleep quality was positively associated with quality of life (β = 0.169, *p* = 0.024). Sleep duration was not significantly associated with quality of life (β = −0.102, *p* = 0.225), and students reporting good health (compared with poor/fair health) did not differ significantly in quality of life (β = 0.077, *p* = 0.302).

Supplementary Table S3 presents the explained variance (R^2^) for both the measurement and structural components of the SEM. The latent constructs explained a substantial proportion of the variance in their respective indicators, with R^2^ values ranging from 0.628 to 0.904 for the WHOQOL-BREF domains and from 0.783 to 0.848 for the DASS-21 subscales, indicating strong measurement properties. For the structural model, the included predictors explained 20.5% of the variance in quality of life.

The structural model explained approximately one-fifth of the variance in quality of life, suggesting that demographic characteristics, self-rated health, and lifestyle behaviors are important correlates of students’ well-being (Table 4). However, the relatively modest R^2^ values also indicate that quality of life is influenced by additional factors not included in the present model, such as academic stress, social support, dietary behaviors, resilience, personality characteristics, and environmental factors. Future studies incorporating these variables may improve the explanatory performance of structural models examining student well-being.

## 4. Discussion

### 4.1. Principal Findings

Our study examined the structural associations among demographic characteristics, lifestyle behaviors, self-rated health, and quality of life among undergraduate students. Our findings showed that quality of life is associated with multiple interrelated behavioral and health-related factors rather than with demographic characteristics alone. We found that excellent self-rated health and better sleep quality exhibited the strongest positive associations with quality of life. The findings also demonstrated that there was a sex difference related to lifestyle behaviors, including sleep quality, sleep duration, and physical activity. However, age, living arrangement, financial support, and most academic characteristics did not show statistically significant direct associations with lifestyle behaviors or quality of life.

### 4.2. Comparison with Previous Studies

Our findings of a strong association between excellent self-rated health and quality of life were consistent with previous studies [20,21]. Self-rated health reflects an individual’s holistic perception of physical functioning, psychological well-being, social participation, and functional capacity, and therefore captures aspects of health that may not be fully represented by objective clinical indicators. In our study, we also found that students reporting very good or excellent health demonstrated substantially higher quality of life scores than those reporting poor or fair health. This can be related to several mechanisms. Students who perceive themselves as healthier are more likely to engage in positive health behaviors, maintain greater participation in academic and social activities, experience fewer physical limitations, and demonstrate greater resilience when confronted with academic stress [22]. Better perceived health may also promote greater confidence, optimism, and life satisfaction, which contribute directly to psychological and social dimensions of quality of life [23]. Conversely, students reporting poorer health may experience reduced energy, chronic symptoms, or emotional concerns that interfere with daily functioning and negatively influence their overall life satisfaction [3].

In our study, there was a significant positive association between better sleep quality and quality of life, whereas sleep duration itself was not independently associated with quality of life after adjustment for other variables. These findings suggest that the subjective experience of restorative sleep may be more influential for students’ well-being than the number of hours slept alone. This is because sleep quality represents a multidimensional construct encompassing sleep continuity, restfulness, sleep efficiency, and satisfaction with sleep [24]. High-quality sleep contributes to cognitive performance, emotional regulation, memory consolidation, immune function, and academic productivity, whereas poor sleep has consistently been associated with fatigue, impaired concentration, emotional instability, anxiety, depression, and reduced life satisfaction [12,25]. University students are particularly susceptible to poor sleep because of irregular study schedules, prolonged screen exposure, social commitments, and academic demands, all of which may compromise healthy sleep patterns [26,27].

Although sleep quality and sleep duration are often considered together, they represent distinct dimensions of sleep health. Sleep duration quantifies the amount of sleep obtained, whereas sleep quality reflects broader characteristics including sleep continuity, efficiency, restfulness, and perceived satisfaction. Individuals obtaining similar sleep durations may differ substantially in sleep fragmentation, nocturnal awakenings, or restorative sleep. Consequently, sleep quality may provide a more comprehensive representation of sleep health, which could explain its stronger association with quality of life observed in the present study.

We found that sex-related differences in health behaviors persist even after accounting for other demographic characteristics. Compared with female students, male sex was associated with poorer sleep quality, shorter sleep duration, and lower levels of physical activity. The observed differences in sleep behaviors may reflect variations in daily routines, lifestyle habits, academic engagement, recreational activities, and health-seeking behaviors between male and female students. Male students may be more likely to engage in prolonged electronic media use, late-night gaming, irregular sleep schedules, or other behaviors that compromise sleep quality and reduce total sleep duration. Sex-specific differences in sleep-related behaviors have also been reported among young adults. Sulis et al. found sex-specific associations between sleep routines and somatic health indicators in a sample of 962 young adults aged 18–30 years, highlighting that the relationships between sleep behaviors and health may differ by sex [28]. By contrast, some studies showed that male students have high-quality sleep compared to female ones [29,30].

Our study found that a GPA of 4.50 or higher was significantly associated with physical activity compared to students with lower academic performance. This finding contributes to growing evidence suggesting that regular physical activity may coexist with strong academic achievement rather than compete with it [31]. This can be related to several reasons. Regular physical activity has been shown to improve cerebral blood flow, executive functioning, attention, working memory, and stress regulation, all of which are important for academic success [32]. Exercise may also reduce symptoms of depression and anxiety while enhancing sleep quality and emotional well-being, thereby indirectly supporting learning and academic performance.

### 4.3. Strengths and Limitations

This study has several strengths. We employed structural equation modeling, which helps us to simultaneously examine multiple interrelated associations while accounting for measurement error in the latent constructs of psychological distress and quality of life. Compared with conventional regression analyses, SEM provides a more comprehensive understanding of the complex relationships among demographic characteristics, lifestyle behaviors, and health outcomes. Validated and internationally recognized instruments, including the DASS-21 and WHOQOL-BREF, were used to assess psychological distress and quality of life, enhancing the reliability and comparability of the findings with previous studies. We examined multiple dimensions of student health simultaneously, including sleep quality, sleep duration, physical activity, self-rated health, and demographic characteristics, providing a holistic perspective on factors associated with quality of life among university students.

However, several limitation should also be considered. The use of a cross-sectional design precludes conclusions regarding temporal or causal relationships. Although SEM allows for examination of complex structural associations, the observed relationships represent statistical associations rather than causal pathways. Longitudinal studies are therefore required to establish the temporal sequence among lifestyle behaviors, psychological distress, and quality of life. Our study relied exclusively on self-reported data, which may be influenced by recall bias, social desirability bias, and subjective interpretation. Measures such as sleep quality, sleep duration, and physical activity were based on participants’ perceptions rather than objective assessments. Those measures were also assessed using single-item measures. Although these questions provided a practical and low-burden approach for assessing key lifestyle behaviors in the student survey, single-item measures may have limited reliability and may not adequately capture the multidimensional nature of sleep and physical activity. Future studies should use validated multi-item instruments, such as comprehensive sleep and physical activity questionnaires, and, where feasible, objective measures including actigraphy or wearable devices to improve measurement precision. Participants were recruited using convenience sampling from public universities in Riyadh. Consequently, the findings may not be generalizable to students attending private universities, institutions located in other regions of Saudi Arabia, or university populations in different countries. Students who voluntarily participated through QR-code recruitment may also have differed systematically from those who chose not to participate, introducing potential selection bias. Although this study considered several important demographic and behavioral variables, other factors that may influence quality of life, including dietary habits, body mass index, social support, academic workload, internet use, substance use, and pre-existing chronic medical conditions, were not assessed. These variables may act as potential confounders or mediators and should be incorporated into future research. The final sample included 198 participants, which is slightly below the commonly cited heuristic threshold of 200 for SEM. No a priori power calculation was performed before data collection. Although the final model converged successfully and demonstrated acceptable overall fit, model fit alone does not establish that this study was adequately powered to detect all associations of interest. The relatively modest sample may have limited the statistical power to detect small effects, particularly for predictors represented by multiple categorical contrasts. Larger studies with prospective sample-size or power calculations are needed to confirm the observed associations and provide more precise parameter estimates.

## 5. Conclusions

This study demonstrated that quality of life among undergraduate students was primarily associated with self-rated health and sleep quality. Students reporting excellent health and better sleep quality experienced higher quality of life, whereas demographic characteristics showed more limited associations after adjustment for other factors. These findings highlight the importance of promoting healthy sleep and supporting students’ overall health as part of university health-promotion initiatives. Because this study employed a cross-sectional design, the observed relationships should be interpreted as associations rather than causal effects. Future longitudinal studies are warranted to confirm these findings and to identify additional factors influencing quality of life among university students.

## Figures and Tables

**Table 1:** Sociodemographic, academic, lifestyle, and psychological characteristics of the study participants (N = 198).

Characteristic	N = 198 *n* (%)
Age (years)	
18–20	44 (22.2%)
21–23	114 (57.6%)
≥24	40 (20.2%)
Sex	
Male	105 (53.0%)
Female	93 (47.0%)
Living arrangement	
Family	118 (59.6%)
University housing	68 (34.3%)
Other (friends or alone)	12 (6.1%)
Academic year	
Year 1	19 (9.6%)
Year 2	46 (23.2%)
Year 3	87 (43.9%)
Year 4/internship	46 (23.2%)
Grade-point average (GPA)	
<3.50	61 (30.8%)
3.50–4.49	97 (49.0%)
4.50–5.00	40 (20.2%)
Self-rated health status	
Poor/fair	42 (21.2%)
Good	100 (50.5%)
Very good/excellent	56 (28.3%)
Sleep quality	
Poor	53 (26.8%)
Fair	83 (41.9%)
Good	62 (31.3%)
Average sleep duration (hours/night)	
<5 h	30 (15.2%)
5–7 h	108 (54.5%)
>7 h	60 (30.3%)
Physical activity	
Never	30 (15.2%)
1–2 times/week	98 (49.5%)
≥3 times/week	70 (35.4%)
Part-time employment	
Yes	53 (26.8%)
No	145 (73.2%)
Learning modality	
Face-to-face	169 (85.4%)
Blended	16 (8.1%)
Online	13 (6.6%)
Continuous variables, Mean (SD)	
Depression score (DASS-21)	16.0 (8.0)
Anxiety score (DASS-21)	16.0 (8.0)
Stress score (DASS-21)	17.0 (7.0)
WHOQOL-BREF Physical Health domain	11.81 (2.29)
WHOQOL-BREF Psychological domain	11.63 (2.57)
WHOQOL-BREF Social Relationships domain	10.60 (3.80)
WHOQOL-BREF Environment domain	11.30 (3.10)

**Table 2:** Model fit indices for the final structural equation model.

Fit Index	Recommended	Observed
χ^2^ (df)	—	168.13 (84)
χ^2^/df	<3.0	2.00
CFI	≥0.90	0.937
TLI	≥0.90	0.914
RMSEA (90% CI)	<0.08	0.071 (0.055–0.087)
SRMR	<0.08	0.070

**Table 3:** Standardized structural path coefficients of the final SEM.

Outcome	Predictor	Standardized β	95% CI	*p*-Value
Sleep quality	Age (Ref: 18–20 years)			
	21–23 years	−0.085	(−0.338, 0.167)	0.346
	≥24 years	0.002	(−0.339, 0.343)	0.983
	Sex (Ref: Female)			
	Male	−0.405	(−0.579, −0.231)	<0.001
	Living arrangement (Ref: Family)			
	University hostel	0.014	(−0.203, 0.231)	0.847
	Other residence	0.035	(−0.421, 0.491)	0.630
	GPA (Ref: <3.50)			
	3.50–4.49	0.047	(−0.106, 0.200)	0.547
	≥4.50	0.024	(−0.190, 0.238)	0.783
	Financial support (Ref: High)			
	Medium	−0.035	(−0.235, 0.165)	0.655
	Low	0.022	(−0.185, 0.229)	0.764
Sleep duration	Age (Ref: 18–20 years)			
	21–23 years	−0.048	(−0.245, 0.149)	0.609
	≥24 years	0.001	(−0.266, 0.268)	0.991
	Sex (Ref: Female)			
	Male	−0.208	(−0.381, −0.035)	0.019
	Living arrangement (Ref: Family)			
	University hostel	0.095	(−0.062, 0.252)	0.235
	Other residence	−0.114	(−0.232, 0.004)	0.056
	GPA (Ref: <3.50)			
	3.50–4.49	0.074	(−0.093, 0.241)	0.385
	≥4.50	0.148	(−0.036, 0.332)	0.115
	Financial support (Ref: High)			
	Medium	−0.087	(−0.236, 0.062)	0.252
	Low	0.120	(−0.014, 0.254)	0.078
Physical activity	Age (Ref: 18–20 years)			
	21–23 years	0.041	(−0.132, 0.214)	0.639
	≥24 years	0.057	(−0.111, 0.225)	0.504
	Sex (Ref: Female)			
	Male	−0.299	(−0.466, −0.132)	<0.001
	Living arrangement (Ref: Family)			
	University hostel	−0.044	(−0.214, 0.126)	0.589
	Other residence	−0.081	(−0.271, 0.109)	0.319
	GPA (Ref: <3.50)			
	3.50–4.49	0.137	(−0.018, 0.292)	0.083
	≥4.50	0.182	(0.021, 0.343)	0.027
	Financial support (Ref: High)			
	Medium	0.091	(−0.036, 0.218)	0.159
	Low	−0.041	(−0.186, 0.104)	0.582
Quality of life	Health status (Ref: Poor/Fair)			
	Good	0.077	(−0.069, 0.223)	0.302
	Very good/Excellent	0.492	(0.306, 0.678)	<0.001
	Sleep quality	0.169	(0.022, 0.316)	0.024
	Sleep duration	−0.102	(−0.266, 0.062)	0.225

**Table 4:** Explained variance in endogenous variables in the structural model.

Endogenous Variable	R^2^	Variance Explained (%)
Sleep quality	0.155	15.5
Sleep duration	0.039	3.9
Physical activity	0.115	11.5
Quality of life	0.205	20.5

## Data Availability

The datasets used in this study are available from the first author on reasonable request. The data are not publicly available due to privacy and ethical restrictions.

## Supplementary Materials

The following supporting information can be downloaded at: https://www.mdpi.com/article/10.3390/healthcare14162528/s1, Table S1: Internal consistency reliability of the DASS-21 and WHOQOL-BREF domains (N = 198); Table S2: Reliability and convergent validity of the latent constructs; Table S3: Explained variance (R^2^) in endogenous variables.
