# Supplementary material for: Associations Among Demographic Characteristics, Sleep Behaviors, Physical Activity, Health Status, and Quality of Life Among Undergraduate Students: A Structural Equation Modeling Study
Source: Healthcare (Basel). 2026 Aug 13;14(16):2528. doi: 10.3390/healthcare14162528 (PMC13512063; doi:10.3390/healthcare14162528)
Supplement: Supplementary file 1 [file healthcare-14-02528-s001.zip › healthcare-4504819-supplementary.pdf]

Supplementary materials

Associations Among Demographic Characteristics, Sleep Behaviors, Physical Activity, Health Status, and Quality of Life Among Undergraduate Students: A Structural Equation Modeling Study

Bandar S. Alharbi and Majed M. Aljabri *

Community and Psychiatric Mental Health Nursing Department, College of Nursing, King Saud University, Riyadh 12375, Saudi Arabia; banalharbi@ksu.edu.sa

* Correspondence: amajad@ksu.edu.sa

Table S1. Internal consistency reliability of the DASS-21 and WHOQOL-BREF domains (N = 198).

Instrument / Domain	No. of items	Cronbach's α	McDonald's ω
**DASS-21**			
Depression	7	0.81	0.86
Anxiety	7	0.78	0.85
Stress	7	0.75	0.83
**WHOQOL-BREF**			
Physical Health	7	0.74	0.85
Psychological Health	6	0.72	0.83
Social Relationships	3	0.80	0.81
Environment	8	0.87	0.90

Table S2. Reliability and convergent validity of the latent constructs

Construct	Indicator	Standardized Loading (λ)	Cronbach's α	Composite Reliability (CR)	AVE
Mental Distress			0.928	0.929	0.814
	Depression	0.885			
	Anxiety	0.921			
	Stress	0.901			
Quality of Life			0.911	0.929	0.778
	Physical Health	0.794			
	Psychological Health	0.825			
	Social Relationships	0.888			
	Environmental Health	0.952			

Table S3. Explained variance (R^2) of endogenous variables

**Outcome**	**R^2**
Depression	0.783
Anxiety	0.848
Stress	0.812
Physical health	0.628
Psychological health	0.679
Social relationships	0.785
Environmental health	0.904
Sleep quality	0.155
Sleep duration	0.039
Physical activity	0.115
Quality of life	0.205
